# Transcatheter Aortic Valve Replacement in Patients at High Risk of Coronary Obstruction

**DOI:** 10.1016/j.jscai.2022.100347

**Published:** 2022-06-22

**Authors:** Yousif Ahmad, Luke Oakley, Sunghan Yoon, Danon Kaewkes, Tarun Chakravarty, Chinar Patel, Tullio Palmerini, Antonio G. Bruno, Francesco Saia, Luca Testa, Francesco Bedogni, Alaide Chieffo, Matteo Montorfano, Antonio L. Bartorelli, Italo Porto, Eberhard Grube, Georg Nickenig, Jan-Malte Sinning, Marco De Carlo, Anna Sonia Petronio, Marco Barbanti, Corrado Tamburino, Alessandro Iadanza, Francesco Burzotta, Carlo Trani, Chiara Fraccaro, Giuseppe Tarantini, Tiziana C. Aranzulla, Giuseppe Musumeci, Giulio G. Stefanini, Maurizio Taramasso, Hyo-Soo Kim, Pablo Codner, Ran Kornowski, Francesco Pelliccia, Luigi Vignali, Raj R. Makkar

**Affiliations:** a Yale School of Medicine, Yale University, New Haven, Connecticut; b Smidt Heart Institute, Cedars-Sinai Medical Center, Los Angeles, California; c Polo Cardio-Toraco Vascolare, Policlinico S. Orsola, Bologna, Italy; d Coronary Revascularisation Unit, IRCCS Policlinico S. Donato, S. Donato Milanese, Italy; e Interventional Cardiology Unit, San Raffaele Scientific Institute, Milan, Italy; f Centro Cardiologico Monzino, IRCCS, University of Milan, Milan, Italy; g Cardiovascular Unit, Department of Internal Medicine and Specialties and IRCCS Ospedale Policlinico San Martino, University of Genova, Genova, Italy; h Department of Medicine II, Heart Center Bonn, University Hospital Bonn, Bonn, Germany; i Azienda Ospedaliero–Universitaria Pisana, Pisa, Italy; j Division of Cardiology, Policlinico-Vittorio Emanuele Hospital, University of Catania, Catania, Italy; k Azienda Ospedaliera Universitaria Senese, Policlinico Le Scotte, Siena, Italy; l Department of Cardiology, Institute of Cardiology, Fondazione Policlinico Universitario A. Gemelli IRCCS, Università Cattolica del Sacro Cuore, Rome, Italy; m Department of Cardiac, Thoracic and Vascular Sciences, University of Padua, Padua, Italy; n Interventional Cardiology, Mauriziano Hospital, Torino, Italy; o Cardio Center, Humanitas Research Hospital IRCCS, Milan, Italy; p Heart Valve Clinic, University Hospital of Zurich, Zurich, Switzerland; q Department of Internal Medicine and Cardiovascular Center, Seoul National University Hospital, Seoul, South Korea; r Department of Cardiology, Rabin Medical Center, Petah Tikva, Israel; s Department of Cardiovascular Sciences, La Sapienza University, Rome, Italy; t UO Cardiologia, Azienda Ospedaliero–Universitaria di Parma, Parma, Italy

**Keywords:** Aortic stenosis, coronary obstruction, transcatheter aortic valve replacement

## Abstract

**Background:**

Coronary obstruction following transcatheter aortic valve replacement (TAVR) is a life-threatening complication. For patients at elevated risk, it is not known how valve choice is influenced by clinical and anatomic factors and how outcomes differ between valve platforms. For patients at high risk of coronary obstruction, we sought to describe the anatomical and clinical characteristics of patients treated with both balloon-expandable (BE) and self-expanding (SE) valves.

**Methods:**

This was a multicenter international registry of patients undergoing TAVR who are considered to be at high risk of coronary obstruction and receiving pre-emptive coronary protection.

**Results:**

A total of 236 patients were included. Patients receiving SE valves were more likely to undergo valve-in-valve procedures and also had smaller sinuses of Valsalva and valve-to-coronary distance. Three-year cardiac mortality was 21.6% with SE vs 3.7% with BE valves. This was primarily driven by increased rates of definite or probable coronary occlusion, which occurred in 12.1% of patients with SE valves vs 2.1% in patients with BE valves.

**Conclusions:**

In patients undergoing TAVR with coronary protection, those treated with SE valves had increased rates of clinical and anatomic features that increase the risk of coronary obstruction. These include an increased frequency of valve-in-valve procedures, smaller sinuses of Valsalva, and smaller valve-to-coronary distances. These patients were observed to have increased cardiac mortality compared with patients treated with BE valves, but this is likely due to their higher risk clinical and anatomic phenotypes rather than as a function of the valve type itself.

## Highlights

• Patients undergoing TAVR with SE valves are more likely to have anatomic features denoting high risk of coronary obstruction than patients receiving BE valves.
• Patients receiving SE valves are also more likely to use valve-in-valve procedures than patients receiving BE valves.
• As a result of these clinical and anatomic features of risk, patients treated with SE valves had higher rates of coronary obstruction and cardiac mortality at 3 years; this is due to their higher risk clinical and anatomic phenotypes rather than a function of the valve type itself.

## Central Illustration

**Figure:**
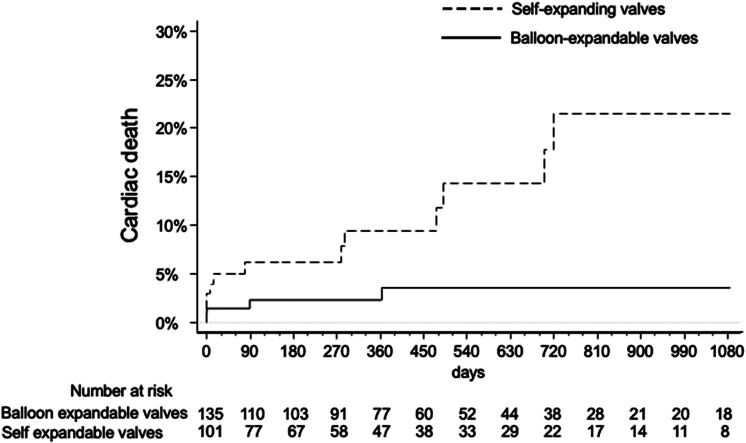

## Introduction

Transcatheter aortic valve replacement (TAVR) is an effective therapy for patients with severe aortic stenosis, with outcomes at least equivalent to surgical aortic valve replacement in randomized clinical trials across the spectrum of surgical risk.^1^, ^2^, ^3^, ^4^, ^5^, ^6^ Iterative advances in procedural planning, implantation technique, and valve technology have led to improved outcomes and reduced procedural complications since the inception of TAVR. There remain potential complications of TAVR, and coronary obstruction is a life-threatening occurrence.^7^, ^8^, ^9^ There are possible interventional techniques to mitigate against the risk of coronary obstruction. These include intentional laceration of the native or bioprosthetic aortic valve leaflets (also known as Bioprosthetic Aortic Scallop Intentional Laceration to prevent Iatrogenic Coronary Artery obstruction [BASILICA])^10^ or pre-emptive wiring of the coronary ostia with or without subsequent stent deployment after valve implantation.^11^^,^^12^ The CORonary PROtection during Transcatheter Aortic Valve Replacement (CORPROTAVR) registry reported outcomes following coronary protection with wiring and possible stenting for patients deemed at high risk of coronary obstruction.^13^ This analysis suggested stent implantation was associated with good survival at 3-year follow-up with low rates of stent thrombosis; wiring only was associated with a risk of delayed coronary occlusion (DCO). It is not known how valve choice is impacted by the risk of coronary obstruction, and there has been no description of outcomes following coronary protection with different valve platforms. We therefore sought to evaluate the 3-year outcomes of patients undergoing TAVR at high risk of coronary obstruction who also underwent coronary protection, describing the features of patients treated with balloon-expandable (BE) and self-expanding (SE) valves and their clinical outcomes.

## Methods

This is a substudy of the CORPROTAVR registry,^13^ in which 236 patients at high risk of coronary obstruction underwent TAVR and were retrospectively analyzed to evaluate the safety and efficacy of coronary protection by preventive coronary wiring and possible eventual stenting across the coronary ostia. This was a multicenter, international registry study involving 19 participating centers worldwide. Data on patients undergoing coronary protection were drawn from general prospective and retrospective databases, which were approved by local ethics committees, with informed consent provided by patients.

The inclusion and exclusion criteria have been previously described,^13^ but in brief, patients considered to be at high risk of coronary obstruction and who underwent coronary protection were included. Patients with true de novo ostial coronary disease were excluded, as were those who had prior ostial stent implantation. Patients had to undergo pre-emptive coronary protection (ie, before valve deployment) to be included.

The current analysis focuses on the characteristics of patients treated with different valve types (BE or SE) and their clinical outcomes.

The height of the coronary ostia in relation to the virtual basal ring or the surgical bioprosthetic sewing ring, the width of the sinus of Valsalva (SOV), and the distance between the virtual transcatheter valve and the protected coronary ostia (VTC) were measured using computed tomography (CT) at each participating center.^8^^,^^14^ A VTC cutoff value ​<4 ​mm was used to analyze the risk of DCO among patients protected with wires only. VTC was defined as the distance between the coronary ostia and the virtual valve frame, measured by positioning a virtual valve during CT reconstruction. The distance between the SOV and the transcatheter heart valve (THV) was measured as the difference between the SOV diameter and THV diameter. A cutoff of less than 3 ​mm was used.

In terms of clinical outcomes, we considered cardiac mortality, all-cause mortality, myocardial infarction (MI), stroke, and the composite of cardiac death, MI, or stroke. Delayed definite coronary occlusion was defined as coronary occlusion documented by coronary angiography or autopsy as occurring any time after wire removal in patients who did not receive stents. In addition, definite or probable coronary occlusion death was defined as any death due to stent thrombosis, DCO, or sudden death. Due to baseline differences in clinical characteristics and anatomy between patients treated with BE and SE valves, no formal statistical group comparisons were performed for these clinical outcomes.

Continuous variables are reported as mean ​± ​standard deviation unless otherwise stated. Continuous variables were compared using the Student *t* test. Categorical variables are reported as counts and percentages and were compared using the chi-square statistic. Event rates were determined using the Kaplan-Meier method. Hazard ratios and 95% confidence intervals were determined using Cox regression models. Multivariate analyses were performed using parsimonious models that included potential confounders unevenly distributed across groups. Two-sided *P* values ​< ​.05 were considered to indicate statistical significance. Statistical analyses were performed using SAS version 9.4 (SAS Institute). TP had full access to all the data in the study and takes responsibility for its integrity and the data analysis.

## Results

Among the 236 patients included in the analyses, 135 patients (57.2%) were treated with BE valves, and 101 (42.8%) were treated with SE valves. Baseline clinical characteristics of patients stratified by the type of valve implanted are reported in Table 1. Patients receiving SE valves were more likely to be undergoing a valve-in-valve (ViV) procedure and had lower coronary heights at baseline; the width of the sinuses was also less with SE valves. The median follow-up duration was 370 ​days (quartiles: 131-716 ​days).

**Table 1:** Baseline clinical and procedural characteristics of patients undergoing coronary protection stratified by the type of valve implanted.

Characteristics	Balloon-expandable valves (*n* ​= ​135)	Self-expanding valves (*n* ​= ​101)	*P* value
Age, y	80.3 ​± ​9.4	80.2 ​± ​7.6	.93
Male sex	53/135 (39.2%)	22/101 (21.7%)	.07
Hypertension	116/135 (85.9%)	77/101 (76.2%)	.82
Body mass index, kg/m^2^	25.9 ​± ​4.8	25.0 ​± ​4.6	.81
Diabetes mellitus	32/135 (23.7%)	16/101 (15.8%)	<.001
Prior myocardial infarction	28/135 (20.7%)	6/101 (5.9%)	.52
Prior PCI	31/135 (23.0%)	19/101 (18.8%)	.51
Prior CABG	18/135 (13.3%)	24/101 (23.7%)	.57
Prior stroke	12/135 (8.9%)	3/101 (2.9%)	.11
Chronic kidney disease	58/135 (43.0%)	40/101 (39.6%)	.70
LVEF, %	55.9 ​± ​12.2	53.9 ​± ​12.2	.21
NYHA class III-IV	115/135 (85.2%)	77/101 (76.2%)	.11
Atrial fibrillation	40/135 (29.6%)	26/101 (25.7%)	.69
Coronary artery disease	74/135 (54.8%)	55/101 (57.8%)	.02
Single vessel disease	24/74 (32.4%)	15/33 (45.5%)	
Double vessel disease	22/74 (29.7%)	14/33 (42.2%)	
Triple vessel disease	18/74 (24.3%)	4/33 (12.2%)	
Peripheral arterial disease	32/135 (23.7%)	17/101 (16.8%)	.38
STS score	8.4 ​± ​8.4	7.0 ​± ​4.4	.12
EuroSCORE II	10.5 ​± ​10.5	10.6 ​± ​8.8	.95
Valve-in-valve procedure	52/135 (38.5%)	71/101 (70.2%)	<.001
Preprocedural AVA, mm^2^	0.7 ​± ​0.4	0.7 ​± ​3.3	.87
Preprocedural MG, mm Hg	40.1 ​± ​17.0	40.5 ​± ​21.6	.86
Postprocedural AVA, mm^2^	1.4 ​± ​3.6	1.2 ​± ​3.8	.26
Postprocedural MG, mm Hg	14.6 ​± ​7.8	16.8 ​± ​9.6	.05
Residual PV leak (moderate or severe)	7/135 (5.2%)	6/101 (5.9%)	.97
Left coronary height, mm	9.6 ​± ​4.1	8.3 ​± ​3.4	.02
Right coronary height, mm	12.2 ​± ​4.6	10.3 ​± ​3.9	.04
Right coronary sinus width, mm	28.1 ​± ​5.4	25.1 ​± ​5.3	.02
Left coronary sinus width, mm	28.9 ​± ​4.6	26.0 ​± ​5.2	.07
Noncoronary sinus width, mm	28.6 ​± ​5.0	25.6 ​± ​5.0	.03

Values are mean ± standard deviation or *n*/*N* (%).

AVA, aortic valve area; CABG, coronary arterty bypass grafting; LVEF, left ventricular ejection fraction; MG, mean gradient; NYHA, New York Heart Association; PCI, percutaneous coronary intervention; PV, paravalvular; STS, Society of Thoracic Surgeons.

A summary of CT measures of the protected coronary arteries stratified by the strategy of coronary protection and the type of valve implanted is shown in Table 2.

**Table 2:** Computed tomographic measures of the protected coronary arteries, stratified by valve type and the strategy of coronary protection.

	Balloon-expandable	Self-expanding	*P* value
Coronary protection with stents			
Protected left coronary artery height	9.1 ​± ​4.5 (68/78 [87.2%])	8.1 ​± ​4.1 (34/41 [83.0%])	.28
Protected right coronary artery height	10.4 ​± ​5.0 (29/31 [93.5%])	9.9 ​± ​4.1 (24/25 [96%])	.69
Any protected coronary artery height	9.5 ​± ​4.7 (97/109 [89.0%])	8.8 ​± ​1.1 (58/66 [87.9%])	.26
Protected left coronary sinus width, mm	28.5 ​± ​5.8 (51/78 [65.3%])	26.1 ​± ​3.7 (9/41 [21.9%])	.23
Protected right coronary sinus width, mm	28.2 ​± ​3.1 (19/31 [61.3%])	25.6 ​± ​3.1 (7/25 [28.0%])	.07
Any protected coronary sinus width, mm	28.4 ​± ​5.2 (70/109 [64.2%])	25.9 ​± ​3.4 (16/66 [24.2%])	.07
VTC	3.9 ​± ​1.6 (89/109 [81.7%])	2.8 ​± ​1.4 (53/66 [80.3%])	<.001
VTC < 4 ​mm	49/89 (55.0%)	43/53 (81.1%)	.003
SOV-THV size	5.8 ​± ​2.8 (60/109 [55.0%])	2.6 ​± ​1.7 (12/66 [18.2%])	<.001
SOV-THV size < 3 ​mm	7/60 (11.7%)	6/12 (50.0%)	.006
Coronary protection with wire (DCO ​+ ​no DCO)			
Protected left coronary artery height	8.3 ​± ​2.5 (25/27 [92.5%])	7.9 ​± ​2.4 (28/28 [100%])	.55
Protected right coronary artery height	9.8 ​± ​3.4 (12/16 [75.0%])	7.6 ​± ​3.1 (15/24 [62.5%])	.09
Any protected coronary artery height	8.8 ​± ​2.9 (37/43 [86.0%])	7.8 ​± ​2.7 (43/52 [82.7%])	.11
Protected left coronary sinus width, mm	29.3 ​± ​4.1 (20/27 [74.1%])	25.1 ​± ​6.2 (17/28 [60.7%])	.01
Protected right coronary sinus width, mm	28.3 ​± ​5.1 (13/16 [81.2%])	28.3 ​± ​4.9 (14/24 [58.3%])	.99
Any protected coronary sinus width, mm	29.0 ​± ​4.5 (33/43 [76.7%])	26.5 ​± ​5.8 (31/52 [59.6%])	.06
VTC	5.2 ​± ​2.3 (34/43 [79.0%])	4.1 ​± ​2.2 (39/52 [75.0%])	.04
VTC < 4 ​mm	8/34 (23.5%)	16/39 (41.0%)	.18
SOV-THV size	6.7 ​± ​4.2 (26/43 [60.5%])	3.9 ​± ​2.7 (18/52 [41.0%])	.02
SOV-THV size < 3 ​mm	5/26 (17.9%)	9/18 (50.0%)	.07
Coronary protection with wire (no DCO)			
Any protected coronary artery height	8.7 ​± ​2.6 (35/41 [85.3%])	7.7 ​± ​2.7 (41/50 [82.0%])	.10
Any protected coronary sinus width	29.0 ​± ​4.5 (31/41 [75.6%])	26.6 ​± ​6.0 (29/50 [58.0%])	.08
VTC	5.3 ​± ​2.2 (32/41 [78.0%])	4.1 ​± ​2.3 (37/50 [74.0%])	.03
VTC < 4 ​mm	6/32 (18.7%)	15/37 (40.5%)	.03
SOV-THV size	4.3 ​± ​6.7 (24/41 [58.5%])	4.3 ​± ​2.6 (16/50 [32.0%])	.99
SOV-THV size < 3 ​mm	3/24 (19.2%)	7/16 (43.7%)	.06
Coronary protection with wire (DCO)			
Any protected coronary artery height	10.5 ​± ​7.2 (2/2 [100%])	8.0 ​± ​0.14 (2/2 [100%])	.67
Any protected coronary sinus width	26 ​± ​0.1 (2/2 [100%])	25.6 ​± ​0.9 (2/2 [100%])	.59
VTC	2.2 ​± ​1.5 (2/2 [100%])	3.9 ​± ​0.1 (2/2 [100%])	.25
VTC < 4 ​mm	2/2 (100%)	1/2 (50%)	.99
SOV-THV size	1.6 ​± ​2.1 (2/2 [100%])	0.9 ​± ​1.1 (2/2 [100%])	.71
SOV-THV size < 3 ​mm	1/2 (50.0%)	2/2 (100%)	.99

All values are measured in units of mm. DCO, delayed coronary occlusion; SOV, sinus of valsalva; THV, transcatheter valve; VTC, the distance between a virtual valve and the ostia of the protected coronary artery. VTC < 4 mm is the number of patients with a VTC distance of less than 4mm. SOH-THV size < 3 mm is the number of patients in whom the difference between the SOV and THV size is less than 3 mm.

Among patients undergoing coronary protection with stents, the use of BE valves was associated with a greater VTC distance (3.9 ​± ​1.6 ​mm for BE vs 2.8 ​± ​1.4 ​mm for SE; *P* < .001). The use of SE valves was also associated with a larger proportion of patients having a VTC distance of less than 4 ​mm (81.1% for SE vs 55.0% for BE; *P* = .003). Similarly, among patients undergoing coronary protection with stents, there was a greater distance between the SOV of the protected coronary and the THV with BE valves than with SE (5.8 ​± ​2.8 ​mm for BE vs 2.6 ​± ​1.7 ​mm for SE; *P* < .001).

Among patients undergoing coronary protection with wiring only, BE valves were associated with a greater protected left coronary sinus width (29.3 ​± ​4.1 ​mm for BE vs 25.1 ​± ​6.2 ​mm for SE; *P* = .01). Similar to patients undergoing coronary protection with stents, BE valves were associated with greater VTC (5.2 ​± ​2.3 ​mm for BE vs 4.1 ​± ​2.2 ​mm for SE; *P* = .04) and greater distance between the SOV of the protected coronary and the THV (6.7 ​± ​4.2 ​mm for BE vs 3.9 ​± ​2.7 ​mm for SE; *P* = .02).

**Figure 1:**
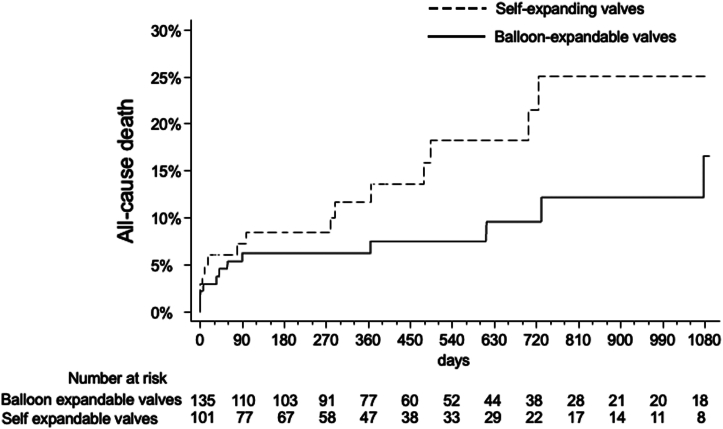
All-cause mortality in patients stratified by the type of valve used (self-expanding or balloon-expandable).

**Figure 2:**
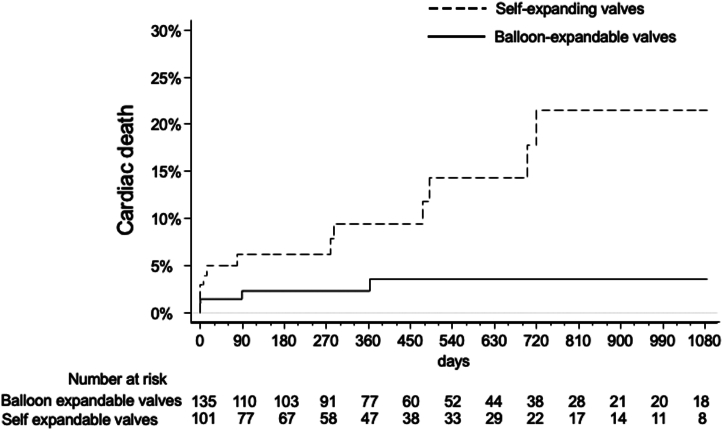
Cardiac death in patients stratified by the type of valve used (self-expanding or balloon-expandable).

**Figure 3:**
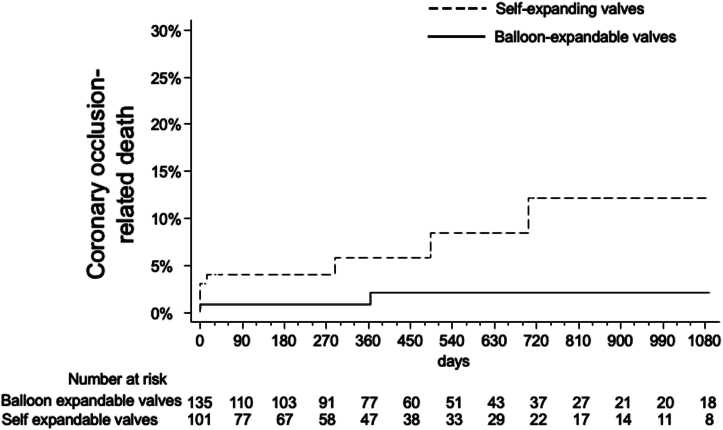
Coronary-occlusion related death in patients stratified by the type of valve used (self-expanding or balloon-expandable).

Three-year outcomes stratified by the type of valve implanted are summarized in Table 3. All-cause mortality results are shown in Figure 1. Three-year cardiac mortality was 21.6% with SE vs 3.7% with BE valves (Figure 2 and Central Illustration). This was primarily driven by increased rates of definite or probable coronary occlusion, which occurred in 12.1% of patients with SE valves vs 2.1% in patients with BE valves (Figure 3). The specific causes of cardiac death in each patient as stratified by the type of valve implanted are shown in Table 4. Of note, all cases but 1 of cardiac death related to definite or probable coronary occlusion with SE valves occurred in ViV procedures.

**Table 3:** Three-year clinical outcomes of patients enrolled in the registry stratified by the type of valve implanted.

	Self-expanding valves	Balloon-expandable valves
All-cause death	15/101 (25.1%)	12/135 (16.6%)
Cardiac death	12/101 (21.6%)	4/135 (3.7%)
Myocardial infarction	4/101 (6.8%)	6/135 (6.9%)
Stroke	4/101 (4.8%)	6/35 (6.2%)
Cardiac death, MI, or stroke	18/101 (28.9%)	14/135 (14.8%)

Values are *n*/*N* (%).

**Central Illustration:**
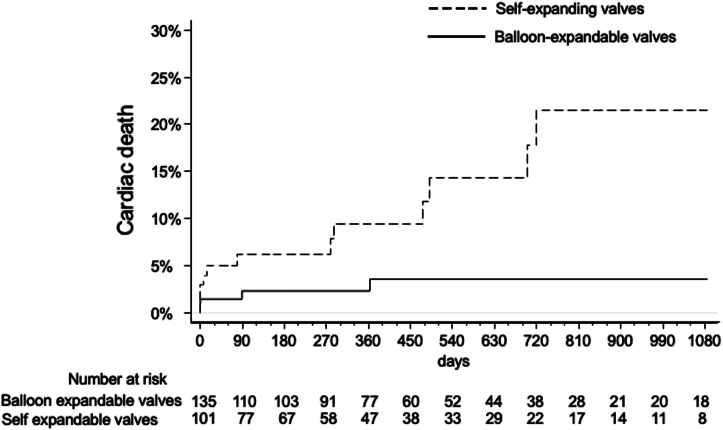
Occurrence of cardiac mortality out to 3-year follow-up in patients treated with self-expanding and balloon-expandable valves. The difference in outcomes reflects the higher risk clinical and anatomical phenotype of patients treated with self-expanding valves rather than as a function of the valve type itself.

**Table 4:** Causes of cardiac death stratified by the type of valve implanted.

Type of transcatheter valve	Age	Cause of cardiac death	Days between TAVR and death	ViV	Type of coronary protection	Stent deployment
Balloon-expandable valves						
SAPIEN XT	83	Heart failure	90	Yes	Stent	LM ​+ ​RCA
SAPIEN 3	82	Sudden death	385	Yes	Stent	LM ​+ ​RCA
SAPIEN 3	91	Procedural complication	0	No	Stent	LM
SAPIEN 3	78	DCO	0	Yes	Wire only	NA
Self-expanding valves						
CoreValve	87	Definite ST	0	Yes	Stent	LM
CoreValve	78	Sudden death	703	Yes	Stent	LM
PORTICO	85	Definite ST	494	Yes	Stent	RCA
CoreValve	79	Sudden death	15	Yes	Wire only	NA
CoreValve	86	DCO	0	No	Wire only	NA
CoreValve	88	Heart failure	8	Yes	Wire only	NA
CoreValve	78	Heart failure	279	Yes	Wire only	NA
CoreValve	84	DCO	0	Yes	Wire only	NA
CoreValve	80	Sudden death	288	Yes	Wire only	NA
Evolut	88	Heart failure	78	Yes	Wire only	NA
Evolut	85	Heart failure	723	No	Wire only	NA
Evolut	83	Unknown	479	No	Wire only	NA

DCO, delayed coronary occlusion; LM, left main; NA, not applicable; RCA, right coronary artery; ST, stent thrombosis; TAVR, transcatheter aortic valve replacement; ViV, valve-in-valve.

There was no significant interaction between the type of transcatheter valve implanted and whether patients did or did not receive stents (*P*_interaction_ ​= ​.38).

A summary of bioprosthetic valves for the ViV cases is shown in Table 5.

**Table 5:** Type of bioprosthetic valve for ViV cases in each group.

Surgical bioprosthesis type	ViV with balloon-expandable valves (*n* ​= ​52)	ViV with self-expanding valves (*n* ​= ​71)
Sorin Mitroflow	28/43 (65.1%)	49/65 (75.4%)
Sorin Freedom SOLO	4/43 (9.3%)	8/65 (12.3%)
Carpentier-Edwards PERIMOUNT Magna Ease	4/43 (9.3%)	5/65 (7.7%)
St. Jude Medical Trifecta	2/43 (4.6%)	2/65 (3.1%)
Sorin Soprano (Sorin Group)	1/43 (2.3%)	0
St. Jude Medical Toronto (St Jude Medical)	3/43 (7.0%)	0
Edwards SAPIEN 3	1/43 (2.3%)	0
Sorin Pericarbon	0	1/65 (1.5%)

Values are *n*/*N* (%).

ViV, valve-in-valve.

## Discussion

The main finding of this study is that among patients deemed at high risk of coronary obstruction and undergoing coronary protection, patients being treated with SE valves had baseline clinical and anatomical features placing them at a higher risk of coronary obstruction. Most significantly, patients being treated with SE valves had a greater frequency of ViV procedures, smaller SOV, and a smaller VTC distance. There are specific reasons, such as the ability to recapture valves before deployment if the initial position is not felt to be optimal, based on which operators may choose SE valves for patients at high risk of coronary obstruction. Furthermore, the use of SE valves in ViV procedures is common due to improved hemodynamics and lower gradients.

The original CORPROTAVR study demonstrated that midterm clinical outcomes following coronary protection and stent implantation were generally favorable, but patients who underwent wiring only without stent implantation were at considerable risk for DCO. To date, that was the largest study of patients at risk for coronary occlusion, and 1 of the only with prolonged follow-up allowing an assessment of clinical outcomes at 3 years. The current study is an exploratory analysis examining the impact of clinical and anatomical factors on the choice to use different valve platforms for patients at high risk of coronary occlusion.

Our study found that the risk of all-cause and cardiac death was numerically greater after the use of SE valves in the context of high risk for coronary obstruction necessitating a coronary protection strategy. This was driven by increased rates of definite or probable coronary occlusion after the use of SE valves. This is consistent with some prior data in the field, with a large French registry of 11,141 patients undergoing TAVR between 2013 and 2015 demonstrating that in-hospital MI related to acute coronary obstruction was 0.4% in patients who received SE valves vs 0.1% in those who received BE valves.^15^ A prior multicenter registry also demonstrated increased rates of delayed coronary obstruction after implantation of SE valves vs BE valves (0.36% vs 0.11%, respectively; *P* ​< ​.01).^9^ Due to the aforementioned inherent differences in baseline clinical and anatomical factors between the 2 groups, these findings in our study are exploratory only and presented with descriptive statistics rather than formal comparisons.

It is noteworthy that in our study the majority of cases of cardiac deaths due to definite or probable coronary occlusion with SE valves occurred in ViV procedures. Indeed, the continuous expansion of the nitinol-based frame and the overfilled SOV may be an underlying mechanism increasing the risk of coronary occlusion-related deaths with SE valves compared to BE devices. However, owing to the supra-annular position of some SE valves, such as the CoreValve/Evolut family, lower gradients have been reported after the ViV procedure with this valve than with BE valves, particularly when surgical bioprostheses having a labeled size ≤21 ​mm are treated.^16^ Poor hemodynamics after ViV in smaller surgical valves have been associated with worse outcomes and a reduction in valve durability and increased valve degeneration. Thus, physicians must balance the potential benefit of SE valves in terms of hemodynamic results against the reported higher risk of coronary flow obstruction.^8^ This risk is particularly high with certain surgically implanted valves (stentless or stented bioprostheses with externally mounted leaflets). Therefore, when ViV is performed with SE valves to treat failed surgical valves with a small labeled size and the aforementioned design, it seems to be a judicious decision to implement a security measure such as placement of an undeployed stent in the coronary artery or other coronary protection methods such as leaflet laceration. Patients undergoing coronary protection with wiring only appeared to have a greater risk of death than those undergoing stenting. The risk of wiring only, without stent deployment, may be increased with SE valves due to continuous expansion of the valve after the end of the procedure. Or this may simply be a reflection of the underlying increased risk of coronary occlusion in patients undergoing ViV procedures. Once again, when performing TAVR in patients at high risk of coronary occlusion, operators must perform a detailed analysis of the CT scan and other patient factors to determine the risk of coronary occlusion and then employ appropriate coronary protection methods (whether that be leaflet laceration or pre-emptive stenting).

In our study, VTC was consistently greater with BE than with SE valves, and this was true irrespective of the type of coronary protection strategy. A greater proportion of patients in the SE group also had a VTC distance of less than 4 ​mm. VTC distance has previously been identified as a risk factor for coronary obstruction.^8^ Similarly, we observed that the mean difference between the SOV and the THV size was greater for BE valves than that for SE valves. It has been demonstrated previously that smaller sized SOV are associated with coronary obstruction, suggesting another potential mechanism for increased coronary obstruction observed with SE valves.^13^ These are known risk factors for iatrogenic coronary occlusion occurring after TAVR in the absence of coronary protection, and therefore, whether these variables are also predictors of adverse events in patients undergoing coronary protection deserves further investigation. Furthermore, 70% of patients in the SE group were undergoing ViV procedures, which are known to have greater risk of coronary occlusion. It is very likely that the adverse outcomes and increased coronary obstruction in the SE group is reflective of these clinical and anatomic factors that placed these patients at elevated risk, rather than a function of the valve platform itself.

Coronary occlusion is a rare event. When new technologies and procedures are adopted into clinical practice, it is incumbent on the community to recognize and understand rare complications as well as common ones. Furthermore, coronary occlusion, whether acute or delayed, carries a dismal prognosis. It is therefore imperative to develop a) strategies to identify patients at risk of this occurrence before we embark on the procedure and b) techniques to mitigate against the risk. To the former, the current study suggests that in patients deemed at high risk of coronary obstruction who underwent coronary protection, patients receiving SE valves were at higher risk of coronary obstruction, due to increased frequency of ViV procedures and high-risk anatomic features such as smaller SOV and a smaller VTC distance.

To the latter point of strategies to mitigate against the risk of coronary occlusion, coronary protection with wires and stents is 1 of the potential interventional techniques. There has also been interest in intentional laceration of the bioprosthetic or native aortic valve leaflet, also known as BASILICA.^10^ In this technique, focused radiofrequency energy is delivered to lacerate the leaflets using electrified guidewires, thereby preventing coronary occlusion after deployment of the TAVR valve. This technique was not performed in the current study, and so our findings do not apply to patients being treated in this way.

### Limitations

This is an observational study with attendant limitations. Patients were not randomized to BE or SE valve types, and so there can be no statements made on the effect of valve type on clinical outcomes. Our results should therefore be considered hypothesis-generating, and we have intentionally provided only descriptive statistics for clinical outcomes rather than formal statistical comparisons or models for adjustment. Furthermore, we have no data regarding factors that influenced the decision of valve choice. Patients receiving SE valves had overall lower coronary heights and were more frequently undergoing ViV procedures. Furthermore, only 50% of patients with a BE valve had VTC <4 ​mm (mean 3.8 ​mm) compared with 81% of patients receiving SE valves (mean VTC = 2.8 mm). All these factors are associated with the risk of coronary obstruction. Only randomization could allow for a true assessment of the impact of SE vs BE valves on outcomes for patients at risk of coronary obstruction. It is clear from the data that patients at higher anatomical risk (based on cardiac CT) and higher clinical risk (based on ViV procedures) were treated with SE valves and that this selection bias likely had a profound impact on clinical outcomes. The retrievability of SE valve platforms may play a role in their selection for cases at high risk of coronary obstruction, and their lower transaortic gradients may play a role in their selection in ViV cases.

In this study, all patients were deemed to be at high risk of coronary obstruction by the individual operators, and no predefined cutoffs or thresholds for risk of coronary obstruction were implemented. Furthermore, we have no certainty that stent deployment truly mitigates the risk of coronary occlusion; this could only be determined by a randomized evaluation of stent deployment vs no stent deployment in patients deemed at high risk of coronary occlusion. There are also potential deleterious downstream effects of stent deployment, such as restenosis or thrombosis. Longitudinal long-term follow-up of patients receiving stents would be required to study this. One potential study design could involve serial CT scans to assess for stent patency and geometrical integrity.

CT analysis was not performed by a single centralized core labortatory in this study. Data regarding other variables such as chronic lung disease or coexistent tricuspid or mitral valve disease were not available. There are also other measurable and unmeasurable confounders that will exert an influence on outcomes; such limitations could only be overcome by randomization. Our findings do not apply to other strategies to prevent coronary occlusion such as leaflet laceration with the BASILICA technique.

## Conclusions

In patients undergoing TAVR with coronary protection, those treated with SE valves had increased rates of clinical and anatomic features that increase the risk of coronary obstruction. These include an increased frequency of ViV procedures, smaller sinuses of Valsalva, and smaller VTC. These patients were observed to have increased cardiac mortality compared with patients treated with BE valves, but this is very likely due to their higher risk clinical and anatomic phenotypes rather than as a function of the valve type itself.

## Declaration of competing interest

Dr Palmerini has received personal fees from Abbott and Edwards Lifesciences. Dr Oakley is a consultant for Edwards Lifesciences. Dr Burzotta received speaker’s fees from Abiomed, Abbott, and Medtronic. Mr Makkar has received research grants from 10.13039/100006520Edwards Lifesciences, 10.13039/100001316Abbott, 10.13039/100004374Medtronic, and 10.13039/100008497Boston Scientific; has served as the national principal investigator for Portico (Abbott) and Acurate (Boston Scientific) U.S. investigation device exemption trials; has received personal proctoring fees from Edwards Lifesciences; and has received travel support from Edwards Lifesciences, Abbott, and Boston Scientific. All other authors declare no conflict of interest.
